# Supplementary material for: MetaRibo-Seq measures translation in microbiomes
Source: Nat Commun. 2020 Jun 29;11:3268. doi: 10.1038/s41467-020-17081-z (PMC7324362; doi:10.1038/s41467-020-17081-z)
Supplement: Supplementary file 10 — Supplementary Data 7 [file 41467_2020_17081_MOESM10_ESM.zip › File2/Confidence_High_Taxonomy/261390_out.krona.html]

Javascript must be enabled to view this page.

members
magnitude
magnitudeUnassigned
count
unassigned
taxon
rank

261390\_out

5

superkingdom
2
5

5
976
phylum

200643
class
5

order
171549
5

family
171552
3

3
genus
838


SRS013800\_contig\_number\_23224
1
species
1947085


SRS098073\_contig\_number\_16906SRS149325\_contig\_number\_15692
species
1947178
2

family
815
2

2
genus
816

246787
species
1

SRS024435\_contig\_number\_31668


SRS049446\_contig\_number\_8743
1
species
817
